# Assessment of Cultivation Factors that Affect Biomass and Geraniol Production in Transgenic Tobacco Cell Suspension Cultures

**DOI:** 10.1371/journal.pone.0104620

**Published:** 2014-08-12

**Authors:** Nikolay Vasilev, Christian Schmitz, Ulrike Grömping, Rainer Fischer, Stefan Schillberg

**Affiliations:** 1 Department Plant Biotechnology, Fraunhofer Institute for Molecular Biology and Applied Ecology IME, Aachen, Germany; 2 Department II–Mathematics, Physics and Chemistry, Beuth University of Applied Sciences, Berlin, Germany; 3 Institute for Molecular Biotechnology, RWTH Aachen University, Aachen, Germany; 4 Institute for Phytopathology and Applied Zoology, Justus-Liebig University Giessen, Giessen, Germany; Mayo Clinic Arizona, United States of America

## Abstract

A large-scale statistical experimental design was used to determine essential cultivation parameters that affect biomass accumulation and geraniol production in transgenic tobacco (*Nicotiana tabacum* cv. Samsun NN) cell suspension cultures. The carbohydrate source played a major role in determining the geraniol yield and factors such as filling volume, inoculum size and light were less important. Sucrose, filling volume and inoculum size had a positive effect on geraniol yield by boosting growth of plant cell cultures whereas illumination of the cultures stimulated the geraniol biosynthesis. We also found that the carbohydrates sucrose and mannitol showed polarizing effects on biomass and geraniol accumulation. Factors such as shaking frequency, the presence of conditioned medium and solubilizers had minor influence on both plant cell growth and geraniol content. When cells were cultivated under the screened conditions for all the investigated factors, the cultures produced ∼5.2 mg/l geraniol after 12 days of cultivation in shaking flasks which is comparable to the yield obtained in microbial expression systems. Our data suggest that industrial experimental designs based on orthogonal arrays are suitable for the selection of initial cultivation parameters prior to the essential medium optimization steps. Such designs are particularly beneficial in the early optimization steps when many factors must be screened, increasing the statistical power of the experiments without increasing the demand on time and resources.

## Introduction

Geraniol is an intermediate metabolite in the monoterpenoid-secoiridoid biosynthesis pathway and is used as a flavor and fragrance compound in the agricultural, food and cosmetic industries. It is an ingredient in 43% of fragrances currently on the market [1]. The annual global demand for geraniol was approximately 327 kg in 2008, with a 3–4% annual market growth rate [2]. The three major geraniol manufacturing strategies are biosynthesis (isolation from essential oils), semi-synthesis (chemical conversion of pinenes) and total synthesis from petrochemicals, and the yield varies between 60% (biosynthesis) and 98% (total synthesis) [2].

The enzyme geraniol synthase (GES) is responsible for the biosynthesis of geraniol from geranyl diphosphate [3]–[10]. GES expression in plants may help to reconstitute the terpenoid-indole alkaloid pathway and therefore satisfy the consumer demand for geraniol.

Plant cell and tissue cultures can be used as an alternative to whole plants for the production of valuable natural compounds [11], [12]. Physical factors that have a major impact on the performance of plant-derived cultures include the inoculum state, size and age [11], [13]–[15], the volume of medium in the culture vessel and the shaking speed, both of which influence on the oxygen supply during cultivation [16], [17]. Biological factors also play an important role, including the use of conditioned medium to influence the accumulation of plant cell biomass [18] and the selection of carbohydrates as sources of energy and carbon due to the low levels of CO_2_ in the closed-glass cultivation vessels during the photoperiod [19]. Light influences both growth and secondary metabolism in plants, e.g. by regulating the diurnal pattern of monoterpenoid synthase transcript levels and monoterpenoid production [20], [21]. Finally, the equilibrium of production may be influenced by introducing excipients such as cyclodextrins that increase the solubility of sparingly-soluble compounds [22].

All the cultivation factors described above may have a direct influence on the productivity of plant cell suspension cultures in terms of biomass accumulation and the synthesis of secondary metabolites. Typically, a one-factor-at-a-time testing approach is used to find optimal cultivation conditions, but this does not identify interactions between factors that can improve or inhibit productivity. Statistical experimental designs are widely used in the biotechnology industry to avoid the drawbacks of testing single factors while leaving the others unchanged. Instead, statistical experimental designs determine the impact of several nutritional and physical factors simultaneously using full factorial, fractional factorial or response surface designs [18], [23]–[25].

To our knowledge, this is the first report describing a statistical experimental design approach that includes multiple physical factors (inoculum size, shaking frequency, filling volume, light) and biological factors (conditioned medium, carbohydrate source and solubilizers) in one experiment. Our results allowed us to optimize geraniol production in transgenic tobacco suspension cultures expressing plastid-targeted GES from *Valeriana officinalis*.

## Materials and Methods

### Origin of transgenic material and initiation of cell suspension cultures

Cloning of *V. officinalis* GES (*VoGES*) cDNA (GenBank accession: KF951406.1) and stable transformation of tobacco plants was conducted as described previously [3]. *N. tabacum* cv. Samsun NN seeds from a homozygous T_7_ line carrying a single *VoGES* expression cassette were selected for initiation of cell suspension cultures. The transgenic seeds were germinated under a 16-h light daily period in sterile conditions on hormone-free MS medium (Duchefa, Netherlands), supplemented with 100 mg/l kanamycin. Next, the intact *in vitro* plants were established on the same medium without antibiotics in plastic transparent containers. Sterile transgenic seedlings were used for the onset of callus culture in Petri dishes containing MS medium with vitamins, supplemented with 0.1 mg/l kinetin and 1 mg/l 1-naphthaleneacetic acid (NAA). Suspended cell cultures were developed subsequently by transferring callus tissue into 10 ml Gamborg's B5 medium plus vitamins, 0.1 mg/l kinetin and 1 mg/l NAA, accommodated in 50 ml TubeSpin bioreactors (Techno Plastic Products AG, Switzerland). Cell suspension cultures were maintained on a gyratory shaker (180 rpm) at 26°C under 16-h illumination (35.6 µmol/s)/8-h darkness and underwent subcultivation every two weeks prior to the start of the screening experimental design. Fresh biomass was collected at the end of the screening cultivation (day 10) and subjected to double filtration under vacuum.

Time curve of *VoGES* suspensions growth was performed in three biological replicates and the samples were collected for analysis every three days after the start of cultivation. Dissolved oxygen was measured online in 250 ml flasks by means of the BPM-60 (BioProcess Monitoring) device (Kühner AG, Switzerland). All the screening or validation experiments were conducted in ISF1-X or in LT-X shaker machines (Kühner AG).

### GC-MS analysis

The extraction and quantitation of geraniol in the plant cell cultures was performed as described earlier by Dong et al. [3] with small modifications. Briefly, plant suspension material was filtrated twice under vacuum and subsequently frozen at −20°C. Two-hundred mg plant cell material was taken, ground and resuspended homogenously in 1 ml citrate-phosphate buffer (pH 5.4), prepared by mixing 27.8 ml 0.2 M dibasic sodium phosphate and 22.2 ml 0.1 M citric acid and topping up to 100 ml with water [26]. The samples were ultra-sonificated for 15 min in a water bath at ambient temperature, followed by the addition of 0.5 ml “Viscozyme L” enzymatic mixture (Sigma-Aldrich, Germany). Next, 1 ml layer of heptane was added to the each sample, containing 10 µg/ml (*Z*)-nerolidol (Sigma-Aldrich) and incubated overnight at 37°C. Next day, each sample was centrifuged at 1750×*g* for 10 min at room temperature. The organic layer was removed and fresh 1 ml heptane was added to sample, vortexed and centrifuged at 1750×*g* for 10 min at room temperature. The last step was repeated once again and all three heptane extracts were pooled, passed through a glass Pasteur pipette provided with a small glass wool plug and ∼1.5 cm anhydrous sodium sulfate (Sigma-Aldrich).

The heptane extract was concentrated under nitrogen gas and the geraniol content was measured quantitatively by a GC-MS machine coupled to a QP2010SE quadrupole mass spectrometer (Shimadzu, Japan). The separation was performed on a 30 m length ×0.25 mm internal diameter Zebron ZB-5 ms column (Phenomenex, USA) containing 0.25 µm stationary phase, preceded by a 5-m guard column. The injected 1 µl aliquot of each sample (split mode 1∶10, injection port temperature 250°C) was subjected to the following temperature program: 45°C for 1 min followed by a gradient of 10°C per min until the temperature reached 300°C, which was held for a further 7 min. The helium inlet pressure was maintained at a constant column flow rate of 1.0 ml/min.

Geraniol was quantified using the base peak (*m/z* = 69) in SIM mode following electric ionization at 1 keV. The recorded mass-spectrum was compared with spectra of a reference standard from the NIST library.

### Experimental design and statistical analysis

The experiment was accommodated in four shaker, satisfying the combinatorial states for the factors light intensity and shaking frequency. Temperature was fixed at 26°C based on our preliminary experiments (Table S1) in order to avoid the necessity for additional shaker machines.

**Figure 1:**
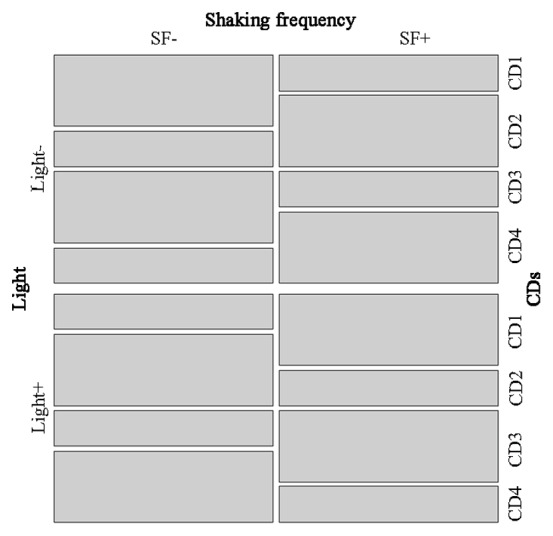
Mosaic plot for three selected factors (light, shaking frequency (SF) and cyclodextrins (CD1-no cyclodextrin; CD2-β-cyclodextrin; CD3-methyl-β-cyclodextrin and CD4-triacetyl-β-cyclodextrin)) showing that any level combination of factors CDs and SF does not determine completely the level of factor “light” which is indicative for limited confounding.

We used an experimental design based on an orthogonal array with 72 runs created with the free open source R package DoE.base [27] (for details, see supplementary material). The design was optimized with respect to confounding of low order effects: all main effects are orthogonal to each other (orthogonal array), confounding between main effects and two-factor interactions was minimized, and in a second step, confounding among two-factor interactions was kept as low as possible. Figure 1 shows a mosaic plot for a triple of design factors that is one of the two triples with the worst-case confounding between main effects and two-factor interactions; even this worst case is reasonably balanced [28].

## Results

Seven cultivation factors (four 2-level factors, two 3-level factors and one 4-level factor) were screened in a randomized experimental design with limited confounding: light, shaking frequency, inoculum size, filling culture volume, addition of conditioned medium, carbohydrate type (sucrose, glucose and D-mannitol) and cyclodextrin type (β-cyclodextrin, methyl-β-cyclodextrin, triacetyl-β-cyclodextrin and no cyclodextrin) (Table 1). Three responses were measured correspondingly: fresh weight, geraniol content and geraniol yield. The full experimental design with 72 runs in coded values and the measured responses are given in Table S2.

**Table 1:** Levels of the screened factors.

Factor	Levels	Abbreviation
Light	11.50 (µmol/cm^2^/s)	Lght-
	35.62 (µmol/cm^2^/s)	Lght+
Shaking frequency	140 rpm	SF-
	180 rpm	SF+
Inoculum size	0.75 g	IS-
	1.40 g	IS+
Filling volume	15 ml	FV-
	20 ml	FV0
	25 ml	FV+
Conditioned medium	100% fresh medium	CM-
	1 ml conditioned medium	CM+
Carbohydrate	Sucrose	Suc
	Glucose	Gluc
	D-Mannitol	Mannit
Cyclodextrin (2 mM)	No cyclodextrin	CD1
	β-cyclodextrin	CD2
	Methyl-β-cyclodextrin	CD3
	Triacetyl-β-cyclodextrin	CD4

The screening experiment was conducted in 50 ml Erlenmeyer shaking flasks.

### Geraniol yield model

**Figure 2:**
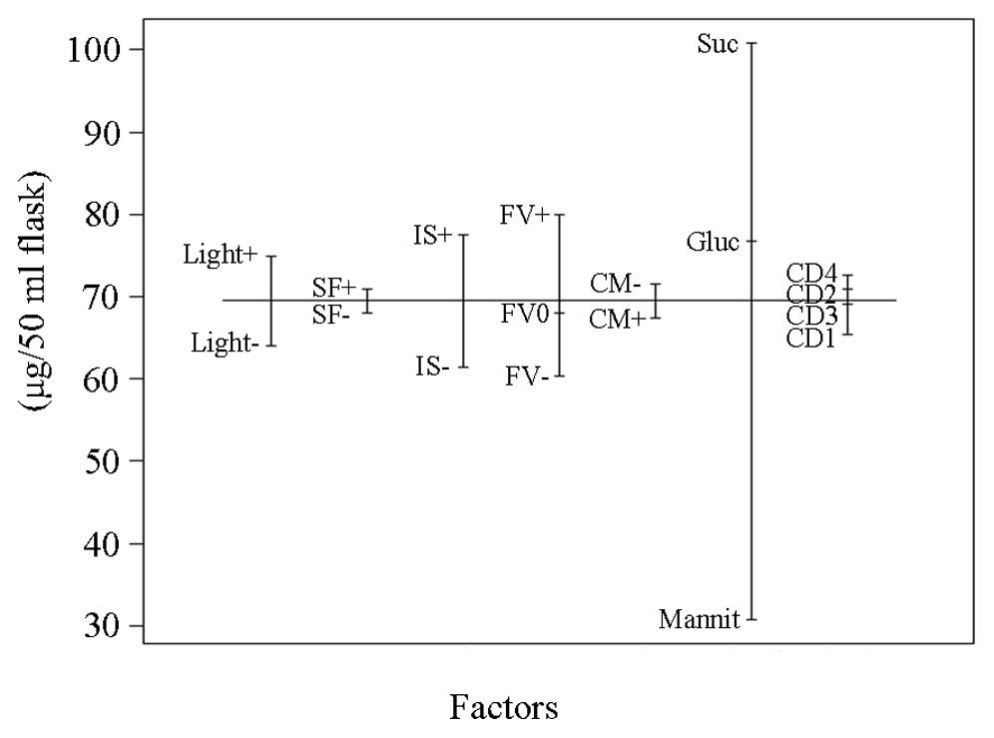
Geraniol yield model plot. The abbreviations are the same as in Table 1.

The productivity of the plant cell platform depends on both the biomass production and the geraniol content per unit biomass. The multiplication of fresh weight by geraniol content in fresh weight stands for geraniol yield in our production system. The geraniol yield model (p<0.05) indicated that the strongest factors with effect on geraniol production are sucrose, filling volume and inoculum size, followed by light (Figure 2).

### Biomass model

**Figure 3:**
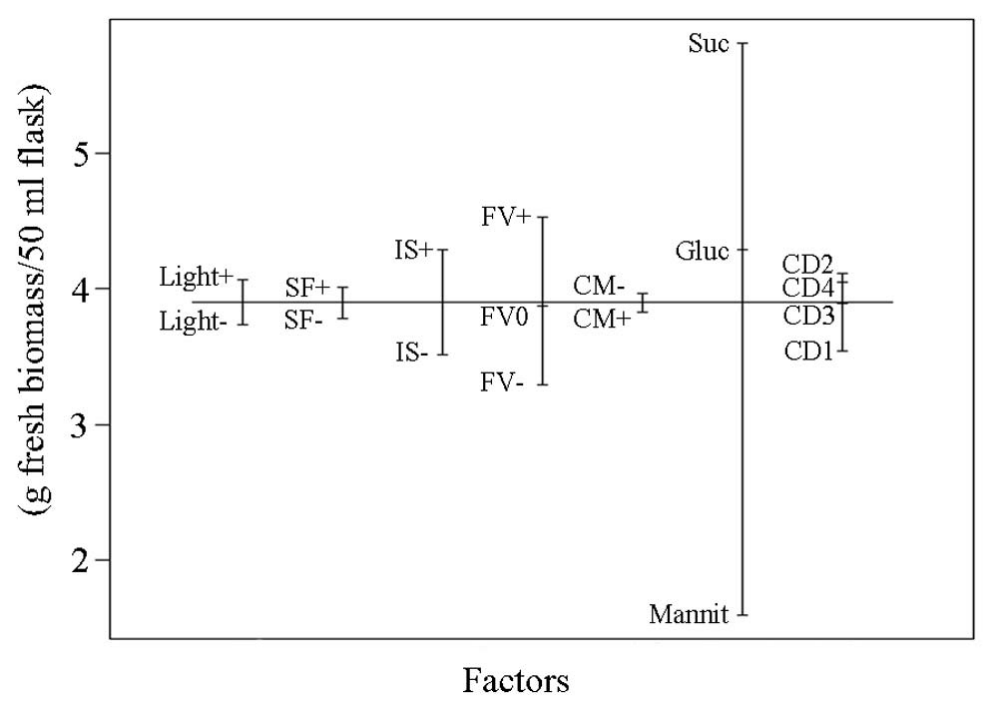
Biomass model plot. The abbreviations are the same as in Table 1.

We created also biomass and geraniol content models (p<0.05) in order to demonstrate how plant cell growth and geraniol content influenced the geraniol yield. The biomass plot showed that the carbohydrate source from all screened factors had the strongest influence on biomass production of the transgenic plant cell cultures (Figure 3). The highest growth was achieved when sucrose was used as a carbon source in the cultivation medium. On the contrary, mannitol considerably reduced the plant cell growth when incorporated as a carbohydrate source into the medium. Filling volume and inoculum size exerted also a positive effect on biomass production but in a less profound extent as sucrose. Therefore, sucrose, filling volume and inoculum size affected biomass production in the same manner as geraniol yield. Shaking frequency, light and addition of conditioned medium and cyclodextrins have exhibited insignificant or negligible effect on the growth of plant cell suspensions.

### Geraniol content model

**Figure 4:**
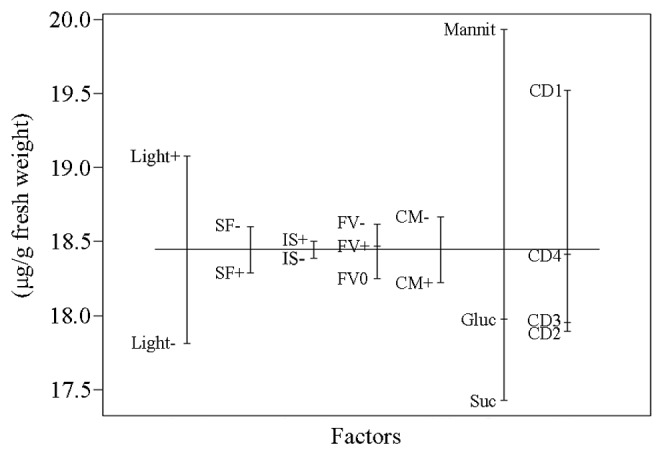
Geraniol content model plot. The abbreviations are the same as in Table 1.

The screening in terms of the geraniol content identified that the use of mannitol led to the highest geraniol levels accumulated in the tobacco transgenic cell cultures (Figure 4). Light had also a potent effect on geraniol biosynthesis. The rest of cultivation factors did not show a strong impact on geraniol production as seen from the geraniol content model (p<0.05).

The pattern of the most significant factors from the biomass model is very similar to the pattern of the strongest factors in the geraniol yield model. This serves as additional evidence that the growth of plant cells contributes to a greater extent to the geraniol yield formation in comparison to the factors acting mainly on the enhancement of geraniol content.

### Significant interactions

**Figure 5:**
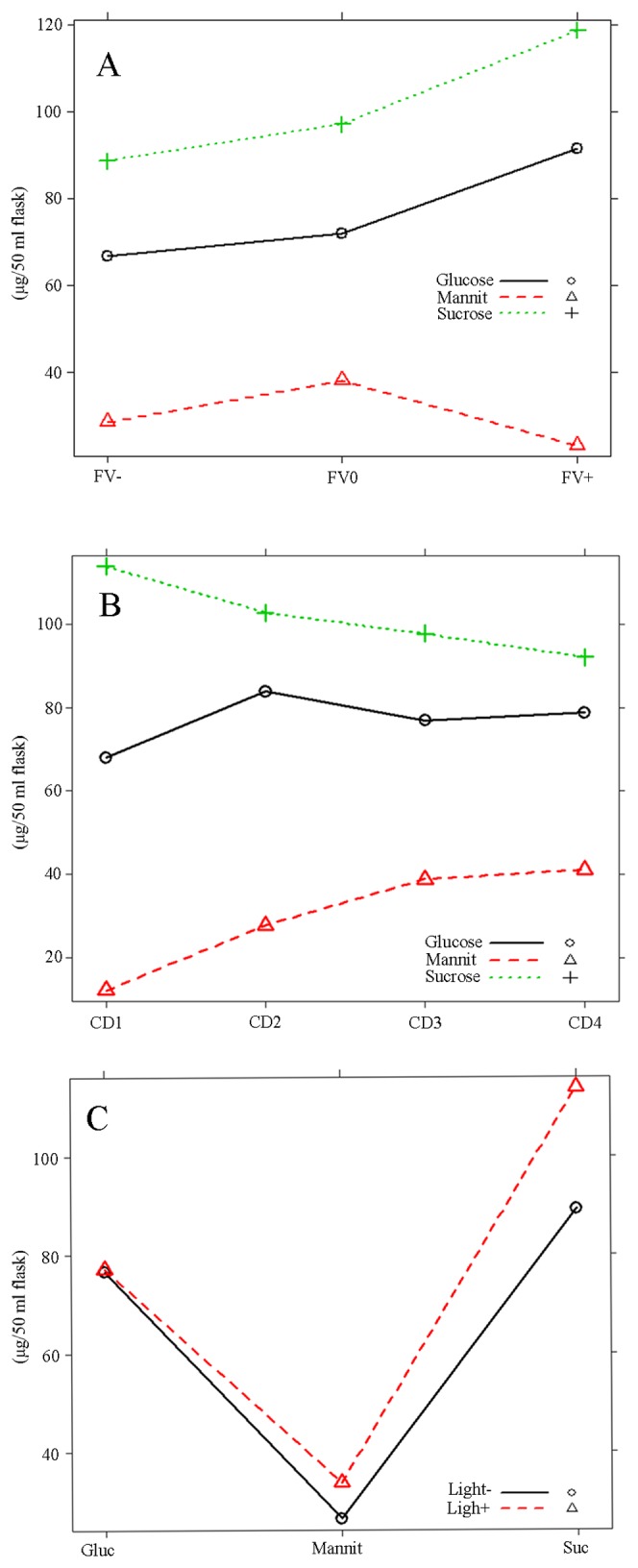
Significant two-factor interactions that affect geraniol yield, detected by the screening design. The abbreviations are the same as in Table 1. (A) Filling volume:sugar interaction. (B) Sugar:cyclodextrins interaction. (C) Light:sugar interaction.

We also identified several potential two-factor interactions concerning geraniol yield (Table S3). The most significant interactions (p<0.01) are filling volume:sugar (Figure 5A), sugar:cyclodextrins (Figure 5B) and light:sugar (Figure 5C). Note that the figures do not show average geraniol yields at each level combination (as a naïve interaction plot would do) but estimated interaction effects from the model with all main effects and 2-factor interactions (obtained with R package effects by Fox 2003 [29]). These are unbiased estimates in the presence of the partial confounding among 2-factor interactions that is present (and in some cases strong) in the experimental design. All three strongest interaction effects involve at least one factor with a strong main effect and are therefore plausible according to the weak effect heredity principle [30]. For two of the strong interactions, both factors have active main effects (strong effect heredity). For instance, Figure 5 shows that an increase of filling volume has a different effect in case mannitol was used as a source of carbohydrate than for the other two sugars. The effect of cyclodextrins on the geraniol yield goes into opposite directions for the sugars mannitol and sucrose, respectively. Light and sucrose potentiate reciprocally their actions on geraniol yield because the corresponding main effects are very strong. Remarkably, shaking frequency also appears to be involved in at least one, possibly several interactions, demonstrating its important role for maximizing geraniol production during plant cell cultivation (Table S3).

### Time-course dynamics

**Figure 6:**
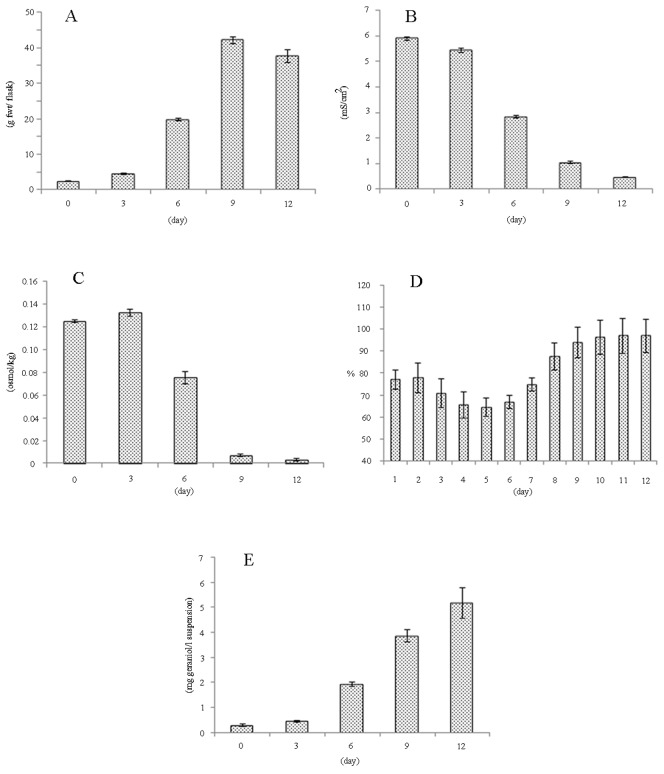
Time-course dynamics. Cultivation conditions: 250 ml shaking flasks; 20 g/l sucrose; 50% filling volume; 5.6% (w/v) inoculum size; 35.62 (µmol/cm^2^/s) light; 2 mM triacetyl-β-cyclodextrin and 180 rpm shaking frequency. (A) Plant cell growth. (B) Electroconductivity. (C) Osmolality. (D) Dissolved oxygen. (E) Geraniol yield.

In the next step, the cultivation of the plant suspension was performed at the favourable levels of factors from the geraniol yield design in order to determine the time dynamics of plant cell productivity in larger shaking flasks (250 ml). We set the screened factors at the beneficial levels in Gamborg's B5-medium supplemented with vitamins and phytohormones in order to keep the corresponding proportions from the geraniol yield design. The following culture conditions were chosen: 20 g/l sucrose; 50% filling volume; 5.6% (w/v) inoculum size; 35.62 µmol/cm^2^/s light; 2 mM triacetyl-β-cyclodextrin and 180 rpm shaking frequency. The suspension cultures grew steadily throughout the cultivation period (Figure 6A) and showed signs of cell death after 14 days post inoculation (dpi). The latter was due to the depletion of nutrients and sucrose in the plant cell cultivation medium which is also seen from the reduction of electroconductivity and osmolality (Figure 6B and 6C). Dissolved oxygen levels started to increase after its minimum at 5 dpi and reached its maximum at 12 dpi, which is indicative for the switch from heterotrophic to autotrophic growth (Figure 6D). The maximal geraniol yield in the plant cell cultures at the end of the cultivation period was 5.12 mg/l (Figure 6E) which is commensurate with the highest levels achieved in the screening design (runs 51 and 60 from Table S2) if these results are extrapolated to the same geraniol yield unit (mg/l).

## Discussion

We initiated a multi-factorial experimental design based on orthogonal arrays to investigate the effects of different cultivation factors on biomass accumulation and geraniol production in transgenic tobacco cell cultures grown in shaking flasks. Such a preliminary screening step is ordinarily needed to define the basic conditions of the key cultivation factors prior the main medium optimization, focused on the sequential manipulation of nutrients and elicitors [25]. Furthermore, cultivation factors are known to demonstrate a variation in a species-dependent manner and thus should be adjusted on a case-by-case basis [18]. Finally, being holistic in nature the optimization based on experimental designs demonstrates improved reproducibility because it takes into account the interactive effects of screened factors [31].

Our study demonstrated that the geraniol yield is mainly dependent on the factors that regulate the growth of the plant cell cultures. The factors acting positively on biomass production had a prevailing influence over the factors that are active on geraniol content with regard to the final geraniol yield. Among the tested cultivation parameters, the carbohydrate source had the strongest effect on biomass formation in the transgenic tobacco cultures. Glucose was utilized in a lesser extent than sucrose and the supplementation of the plant cell cultures with mannitol inhibited strongly their growth. Sucrose is known to be the most universal sugar for the plant cell cultivation, followed by glucose, maltose and raffinose [19], whereas the addition of some sugar alcohols like mannitol may cause osmotic stress in the system [19], [32]. Additionally, these sugar alcohols may serve not only as osmotic agents but they also regulate morphogenesis and metabolism [33]. We observed a similar metabolic effect of mannitol as it was the most potent factor stimulating the geraniol biosynthesis. However, the exact mechanism of induction of geraniol metabolism by mannitol remains to be clarified.

Filling volume is one of the operating parameters affecting the oxygen transfer rate in shaking flasks [34]. Filling volume had also an important effect on biomass production and respectively on geraniol yield of *VoGES* tobacco suspension cultures. Increasing the shaking speed and decreasing the medium volume improved biomass production to a certain extent in shake flask culture of *Atropa belladonna* hairy roots [16]. In our tobacco plant cell cultures, shaking speed did not play a significant role on geraniol yield. This indicates that the tobacco suspension cell cultures might not suffer from serious oxygen limitations at the applied shaking frequency and filling volume levels. Our study showed that shaking frequency may not function as a strong single factor but it is involved in several significant interactions, concerning biomass and geraniol yield mainly, and therefore, it should be also taken into consideration in the initial stage of optimization.

Our experiments identified that inoculum size had a moderate effect whilst the addition of conditioned medium had negligible impact on the biomass production of transgenic tobacco suspensions. Multi-factorial analyses using response surface experimental designs revealed that both inoculum size and conditioned medium had a positive effect on the biomass production in oil palm (*Elaeis guineensis*) cultures [18]. The inoculum size and the addition of conditioned medium had also a beneficial effect on alkaloid production in immobilized *Catharanthus roseus* cells [35]. Previous reports showed that the enhancement of inoculum density usually leads to higher biomass productivity but lower growth rate [13], [15]. The high inoculum size can contribute for the elimination or even diminishment of the lag phase during the cultivation [11]. The experimental design in our investigation could also detect a positive influence of high inoculum levels on geraniol yield. This observation is in agreement with earlier reports in literature showing that several enzymes i.e. geraniol-10-hydroxylase, had increased their activity in high-density cultures of *Catharanthus roseus* [36].

Addition of conditioned medium can enhance the cell density and the accumulation of ginseng polysaccharide in *Panax notoginseng* cell cultures [37]. The lack of potent effect of the conditioned medium in our plant cell cultures may be due to various reasons like reduction of osmolarity, decreasing the sugar and nitrogen concentrations, or to a phytohormone alteration because of the conditioned medium addition, although in general conditioned medium may contain some favourable stimulating factors at the same time [18].

The geraniol content model identified light as an important factor for geraniol biosynthesis. Several studies showed previously that light exerts a strong effect on monoterpenoid metabolism by regulating transcript levels of monoterpenoid synthases, precursor availability, constitutive promoter activity, etc. [20], [21], [38]. Furthermore, the interaction we found between light and sucrose might be also indicative for mixed contribution of both autotrophic and heterotrophic carbon metabolism in cultured tobacco cells [39].

We report herein that transgenic tobacco suspensions expressing *V. officinalis* GES could reach geraniol yield of 5.2 mg/l after 12 days cultivation. This accumulation level in non-fully-optimized conditions is close to the geraniol yield produced by yeast cells expressing the *O. basilium* GES [4]. Additional medium optimization may further improve the geraniol yield and turn transgenic plant cell cultures into an attractive production system of valuable natural products, matching the yields achieved by semi-synthesis and total chemical synthesis. Therefore, a combined, empirical study about optimization of plant suspensions productivity is needed, aiming at both physical and chemical factors and experimental statistical designs may serve as a useful tool.

## Supporting Information

Text S1 **R code for design generation.**
(DOCX)

Table S1 **Preliminary test of temperature effects on biomass and geraniol production.**
(DOCX)

Table S2 **Randomized experimental design for seven independent variables in coded values, and fresh biomass weight, geraniol content and geraniol yield as measured responses.**
(DOCX)

Table S3 **Analysis of Variance table for the model with all main effects and 2-factor interactions.**
(DOCX)
