# Supplementary material for: Assessment of Cultivation Factors that Affect Biomass and Geraniol Production in Transgenic Tobacco Cell Suspension Cultures
Source: PLoS One. 2014 Aug 12;9(8):e104620. doi: 10.1371/journal.pone.0104620 (PMC4130582; doi:10.1371/journal.pone.0104620)
Supplement: Text S1 — R code for design generation. (DOCX) [file pone.0104620.s001.docx]

**Text S1. R code for design generation**

require(DoE.base)

show.oas(factors = list(nlevels=c(2,3,4), number=c(4,2,1)), show = "all")

Experimental.plan <- oa.design(ID=L72.2.43.3.8.4.1.6.1,

nlevels=c(2,2,2,3,2,3,4), columns="min34",

factor.names=list(

Light=c("Lght-","Lght+"),

ShakFreq=c("SF-","SF+"),

InocSize=c("IS-","IS+"),

FilledVol=c("FV-","FV0", "FV+"),

CM=c("CM-","CM+"),

Sugar=c("Suc", "Gluc", "Mannit"),

CDs=c("CD1","CD2","CD3","CD4")),

seed = 9, randomize=TRUE)

Experimental.plan
