# Supplementary material for: Assessment of Cultivation Factors that Affect Biomass and Geraniol Production in Transgenic Tobacco Cell Suspension Cultures
Source: PLoS One. 2014 Aug 12;9(8):e104620. doi: 10.1371/journal.pone.0104620 (PMC4130582; doi:10.1371/journal.pone.0104620)
Supplement: Table S1 — Preliminary test of temperature effects on biomass and geraniol production. (DOCX) [file pone.0104620.s002.docx]

Table S1. Preliminary test of temperature effect on biomass and geraniol production.

| **Temperature** | **Replicates** | **Biomass (g)** | **Mean (g)** | **STDEV (g)** | **Content (µg/g fwt)** | **Mean content (µg/g fwt)** | | **STDEV (µg/g fwt)** |
| --- | --- | --- | --- | --- | --- | --- | --- | --- |
| 20°C | 1 | n.a. |  |  | n.a. |  |  | |
|  | 2 |  |  |  |  |  |  | |
|  | 3 |  |  |  |  |  |  | |
|  | 4 |  |  |  |  |  |  | |
| 26°C | 1 | 2.28 | 2.15 | 0.09 | 19.93 | 17.61 | 1.60 | |
|  | 2 | 2.1 |  |  | 16.62 |  |  |  |
|  | 3 | 2.07 |  |  | 17.40 |  |  |  |
|  | 4 | 2.16 |  |  | 16.47 |  |  |  |
| 32°C | 1 | 1.85 | 1.82 | 0.04 | 16.72 | 17.32 | 0.47 | |
|  | 2 | 1.86 |  |  | 17.27 |  |  |  |
|  | 3 | 1.77 |  |  | 17.40 |  |  |  |
|  | 4 | 1.81 |  |  | 17.87 |  |  |  |

STDEV – standard deviation

n.a. – fresh biomass and geraniol content were not measured due to the very poor and insufficient growth of the plant cells
