# Supplementary material for: Assessment of Cultivation Factors that Affect Biomass and Geraniol Production in Transgenic Tobacco Cell Suspension Cultures
Source: PLoS One. 2014 Aug 12;9(8):e104620. doi: 10.1371/journal.pone.0104620 (PMC4130582; doi:10.1371/journal.pone.0104620)
Supplement: Table S2 — Randomized experimental design for seven independent variables in coded values, and fresh biomass weight, geraniol content and geraniol yield as measured responses. (DOCX) [file pone.0104620.s003.docx]

Table S2. Randomized experimental design for the seven independent variables in coded values, and fresh biomass weight, geraniol content and geraniol yield as measured responses in 50 ml shaking flasks growing *VoGES* plant cells.

| **Run №** | **Light** | **Shaking**  **frequency** | **Inoculum**  **size** | **Filled**  **volume** | **Conditioned**  **medium** | **Carbohydrate** | **CD** | **Biomass (g fwt)** | **Content (µg/g fwt)** | **Yield**  **(µg/flask)** |
| --- | --- | --- | --- | --- | --- | --- | --- | --- | --- | --- |
| 1 | Lght+ | SF+ | IS+ | FV- | CM- | Suc | CD1 | 5.80 | 24.13 | 139.98 |
| 2 | Lght- | SF+ | IS+ | FV0 | CM+ | Gluc | CD2 | 4.97 | 16.96 | 84.28 |
| 3 | Lght+ | SF+ | IS- | FV+ | CM+ | Mannit | CD2 | 1.28 | 21.08 | 26.99 |
| 4 | Lght+ | SF+ | IS- | FV+ | CM- | Suc | CD2 | 6.83 | 17.71 | 120.95 |
| 5 | Lght- | SF- | IS+ | FV- | CM+ | Mannit | CD1 | 0.86 | 21.28 | 18.30 |
| 6 | Lght+ | SF- | IS+ | FV0 | CM- | Gluc | CD3 | 4.09 | 18.86 | 77.14 |
| 7 | Lght+ | SF+ | IS+ | FV0 | CM+ | Mannit | CD3 | 2.39 | 17.08 | 40.81 |
| 8 | Lght+ | SF+ | IS+ | FV- | CM+ | Gluc | CD1 | 4.05 | 17.84 | 72.23 |
| 9 | Lght+ | SF+ | IS+ | FV0 | CM+ | Suc | CD3 | 5.84 | 17.74 | 103.61 |
| 10 | Lght+ | SF- | IS- | FV- | CM- | Gluc | CD4 | 3.38 | 18.08 | 61.11 |
| 11 | Lght+ | SF+ | IS- | FV0 | CM+ | Mannit | CD1 | 0.40 | 24.82 | 9.93 |
| 12 | Lght- | SF- | IS- | FV+ | CM- | Gluc | CD1 | 3.86 | 18.10 | 69.88 |
| 13 | Lght+ | SF- | IS+ | FV- | CM+ | Suc | CD2 | 4.58 | 21.29 | 97.49 |
| 14 | Lght+ | SF- | IS+ | FV+ | CM- | Gluc | CD2 | 6.29 | 17.32 | 108.91 |
| 15 | Lght- | SF+ | IS- | FV- | CM- | Suc | CD1 | 4.85 | 15.50 | 75.17 |
| 16 | Lght- | SF- | IS- | FV+ | CM+ | Mannit | CD4 | 1.25 | 23.14 | 28.92 |
| 17 | Lght- | SF+ | IS+ | FV+ | CM+ | Mannit | CD2 | 2.09 | 18.43 | 38.51 |
| 18 | Lght+ | SF+ | IS+ | FV- | CM- | Gluc | CD2 | 4.26 | 17.75 | 75.62 |
| 19 | Lght- | SF- | IS+ | FV+ | CM+ | Gluc | CD3 | 4.78 | 18.53 | 88.57 |
| 20 | Lght+ | SF+ | IS+ | FV0 | CM- | Suc | CD4 | 6.63 | 17.82 | 118.14 |
| 21 | Lght- | SF- | IS+ | FV0 | CM+ | Mannit | CD1 | 0.77 | 18.79 | 14.47 |
| 22 | Lght+ | SF- | IS+ | FV0 | CM+ | Gluc | CD4 | 4.89 | 18.23 | 89.15 |
| 23 | Lght+ | SF+ | IS- | FV+ | CM- | Gluc | CD3 | 4.53 | 17.69 | 80.11 |
| 24 | Lght+ | SF- | IS- | FV0 | CM+ | Gluc | CD2 | 4.27 | 18.05 | 77.07 |
| 25 | Lght- | SF- | IS- | FV- | CM+ | Suc | CD2 | 3.90 | 15.84 | 61.77 |
| 26 | Lght- | SF+ | IS+ | FV0 | CM- | Gluc | CD1 | 4.15 | 18.73 | 77.74 |
| 27 | Lght- | SF+ | IS+ | FV- | CM+ | Suc | CD4 | 3.95 | 17.12 | 67.63 |
| 28 | Lght+ | SF+ | IS- | FV0 | CM- | Suc | CD1 | 6.92 | 16.86 | 116.68 |
| 29 | Lght+ | SF+ | IS- | FV+ | CM+ | Gluc | CD4 | 5.00 | 16.96 | 84.80 |
| 30 | Lght- | SF- | IS- | FV- | CM- | Mannit | CD1 | 0.37 | 21.79 | 8.06 |
| 31 | Lght- | SF+ | IS- | FV0 | CM+ | Gluc | CD4 | 2.36 | 19.57 | 46.18 |
| 32 | Lght- | SF+ | IS- | FV+ | CM+ | Suc | CD1 | 5.11 | 18.13 | 92.66 |
| 33 | Lght- | SF- | IS+ | FV0 | CM- | Suc | CD2 | 4.69 | 17.38 | 81.50 |
| 34 | Lght+ | SF+ | IS- | FV- | CM- | Mannit | CD3 | 1.20 | 19.57 | 23.49 |
| 35 | Lght+ | SF- | IS- | FV- | CM+ | Mannit | CD2 | 1.76 | 17.98 | 31.65 |
| 36 | Lght+ | SF- | IS- | FV+ | CM+ | Suc | CD3 | 6.21 | 17.03 | 105.76 |
|  |  |  |  |  |  |  |  |  |  |  |
|  |  |  |  |  |  |  |  |  |  |  |
|  |  |  |  |  |  |  |  |  |  |  |
| **Run №** | **Light** | **Shaking**  **frequency** | **Inoculum**  **size** | **Filled**  **volume** | **Conditioned**  **medium** | **Carbohydrate** | **CD** | **Biomass (g fwt)** | **Content (µg/g fwt)** | **Yield**  **(µg/flask)** |
| 37 | Lght- | SF- | IS+ | FV0 | CM+ | Suc | CD1 | 5.63 | 15.71 | 88.43 |
| 38 | Lght+ | SF- | IS- | FV- | CM- | Suc | CD3 | 3.98 | 18.42 | 73.32 |
| 39 | Lght+ | SF+ | IS+ | FV- | CM+ | Mannit | CD3 | 2.31 | 19.38 | 44.76 |
| 40 | Lght- | SF- | IS- | FV0 | CM+ | Mannit | CD3 | 1.86 | 18.41 | 34.25 |
| 41 | Lght- | SF+ | IS- | FV0 | CM- | Gluc | CD3 | 4.22 | 17.93 | 75.68 |
| 42 | Lght- | SF- | IS- | FV- | CM+ | Gluc | CD1 | 2.77 | 17.17 | 47.55 |
| 43 | Lght+ | SF- | IS- | FV+ | CM- | Mannit | CD1 | 0.40 | 23.10 | 9.24 |
| 44 | Lght- | SF+ | IS+ | FV- | CM- | Mannit | CD2 | 1.42 | 18.89 | 26.83 |
| 45 | Lght- | SF+ | IS- | FV- | CM+ | Mannit | CD4 | 1.54 | 17.44 | 26.86 |
| 46 | Lght- | SF- | IS+ | FV- | CM- | Suc | CD3 | 5.03 | 17.40 | 87.53 |
| 47 | Lght- | SF+ | IS+ | FV+ | CM- | Suc | CD2 | 8.70 | 14.41 | 125.38 |
| 48 | Lght+ | SF+ | IS- | FV- | CM+ | Gluc | CD3 | 3.21 | 19.29 | 61.92 |
| 49 | Lght+ | SF+ | IS+ | FV+ | CM+ | Gluc | CD1 | 5.36 | 18.46 | 98.93 |
| 50 | Lght- | SF- | IS+ | FV- | CM+ | Gluc | CD3 | 3.87 | 16.89 | 65.35 |
| 51 | Lght+ | SF- | IS+ | FV+ | CM+ | Suc | CD1 | 7.70 | 18.60 | 143.20 |
| 52 | Lght- | SF+ | IS- | FV+ | CM- | Mannit | CD3 | 1.71 | 17.67 | 30.22 |
| 53 | Lght- | SF- | IS- | FV+ | CM+ | Gluc | CD2 | 4.38 | 16.79 | 73.54 |
| 54 | Lght- | SF+ | IS+ | FV0 | CM- | Mannit | CD4 | 2.24 | 19.61 | 43.92 |
| 55 | Lght+ | SF- | IS- | FV0 | CM- | Gluc | CD1 | 3.79 | 19.35 | 73.35 |
| 56 | Lght+ | SF- | IS+ | FV+ | CM+ | Mannit | CD4 | 3.09 | 18.67 | 57.70 |
| 57 | Lght- | SF+ | IS- | FV0 | CM- | Mannit | CD4 | 1.57 | 17.64 | 27.70 |
| 58 | Lght- | SF+ | IS+ | FV+ | CM- | Gluc | CD4 | 5.43 | 18.45 | 100.19 |
| 59 | Lght- | SF+ | IS- | FV- | CM- | Gluc | CD2 | 3.86 | 17.09 | 65.96 |
| 60 | Lght+ | SF- | IS+ | FV+ | CM- | Suc | CD4 | 7.44 | 19.07 | 141.85 |
| 61 | Lght- | SF- | IS- | FV+ | CM- | Suc | CD4 | 5.87 | 17.13 | 100.53 |
| 62 | Lght- | SF- | IS+ | FV+ | CM- | Mannit | CD3 | 2.65 | 17.51 | 46.39 |
| 63 | Lght+ | SF- | IS- | FV0 | CM+ | Suc | CD4 | 6.14 | 15.85 | 97.34 |
| 64 | Lght- | SF+ | IS- | FV0 | CM+ | Suc | CD2 | 6.32 | 14.80 | 93.56 |
| 65 | Lght+ | SF+ | IS- | FV- | CM+ | Suc | CD4 | 5.19 | 16.53 | 85.78 |
| 66 | Lght- | SF- | IS+ | FV- | CM- | Gluc | CD4 | 5.09 | 17.30 | 88.04 |
| 67 | Lght- | SF- | IS- | FV0 | CM- | Suc | CD3 | 4.40 | 17.52 | 77.08 |
| 68 | Lght+ | SF- | IS- | FV0 | CM- | Mannit | CD2 | 1.68 | 21.89 | 36.78 |
| 69 | Lght+ | SF+ | IS+ | FV+ | CM- | Mannit | CD1 | 0.93 | 23.06 | 21.45 |
| 70 | Lght+ | SF- | IS+ | FV- | CM- | Mannit | CD4 | 1.79 | 22.88 | 40.95 |
| 71 | Lght+ | SF- | IS+ | FV0 | CM- | Mannit | CD2 | 2.64 | 18.38 | 48.52 |
| 72 | Lght- | SF+ | IS+ | FV+ | CM+ | Suc | CD3 | 7.78 | 16.22 | 126.19 |

Legend: SF - shaking frequency; IS - inoculum size; FV - filling culture volume; CM – conditioned medium; Suc – sucrose; Gluc – glucose; Mannit - D-mannitol; CD1 - no cyclodextrin; CD2 - β-cyclodextrin; CD3 - methyl-β-cyclodextrin and CD4 - triacetyl-β-cyclodextrin. For more details about the factor levels, s. Table 1 from the main article body.
