# Supplementary material for: Assessment of Cultivation Factors that Affect Biomass and Geraniol Production in Transgenic Tobacco Cell Suspension Cultures
Source: PLoS One. 2014 Aug 12;9(8):e104620. doi: 10.1371/journal.pone.0104620 (PMC4130582; doi:10.1371/journal.pone.0104620)
Supplement: Table S3 — Analysis of Variance table for the model with all main effects and 2-factor interactions. (DOCX) [file pone.0104620.s004.docx]

Table S3. Analysis of Variance table for the model with all main effects and 2-factor interactions (obtained from the full model using function Anova of R package ‘**car**’ by Fox and Weisberg 2011)

Anova Table (Type II tests)

Response: yield

|  | **Sum of squares** | **Degrees of freedom** | **F-value** | **Pr(>F)** |  |
| --- | --- | --- | --- | --- | --- |
| Light | 1878 | 1 | 46.6562 | 4,57E-02 | *** |
| ShakFreq | 68 | 1 | 1.6777 | 0.2243346 |  |
| InocSize | 3233 | 1 | 80.3351 | 4,29E-03 | *** |
| FilledVol | 3578 | 2 | 44.4429 | 1,06E-02 | *** |
| CM | 220 | 1 | 5.4752 | 0.0413451 | * |
| Sugar | 57484 | 2 | 714.11 | 1,63E-08 | *** |
| CDs | 353 | 3 | 2.9229 | 0.0864829 | . |
| Light:ShakFreq | 2 | 1 | 0.0571 | 0.8159367 |  |
| Light:InocSize | 1 | 1 | 0.0339 | 0.8576106 |  |
| Light:FilledVol | 172 | 2 | 2.1405 | 0.1683517 |  |
| Light:CM | 220 | 1 | 5.4725 | 0.0413863 | * |
| Light:Sugar | 830 | 2 | 10.3131 | 0.0037114 | ** |
| Light:CDs | 482 | 3 | 3.9939 | 0.0415096 | * |
| ShakFreq:InocSize | 46 | 1 | 1.1551 | 0.3077249 |  |
| ShakFreq:FilledVol | 34 | 2 | 0.4237 | 0.6658189 |  |
| ShakFreq:CM | 146 | 1 | 3.6222 | 0.0861671 | . |
| ShakFreq:Sugar | 438 | 2 | 5.4402 | 0.0251946 | * |
| ShakFreq:CDs | 390 | 3 | 3.2267 | 0.0694994 | . |
| InocSize:FilledVol | 163 | 2 | 2.0305 | 0.1819335 |  |
| InocSize:CM | 173 | 1 | 4.2970 | 0.0649647 | . |
| InocSize:Sugar | 52 | 2 | 0.6438 | 0.5457610 |  |
| InocSize:CDs | 269 | 3 | 2.2273 | 0.1477474 |  |
| FilledVol:CM | 109 | 2 | 1.3594 | 0.3004577 |  |
| FilledVol:Sugar | 1857 | 4 | 11.5346 | 0.0009163 | *** |
| FilledVol:CDs | 456 | 6 | 1.8873 | 0.1788443 |  |
| CM:Sugar | 147 | 2 | 1.8217 | 0.2115436 |  |
| CM:CDs | 285 | 3 | 2.3594 | 0.1329303 |  |
| Sugar:CDs | 1914 | 6 | 7.9260 | 0.0024338 | ** |
| Residuals | 402 | 10 |  |  |  |

Significance codes: ‘***’ 0.001, ‘**’ 0.01, ‘*’ 0.05, ‘.’ 0.1, ‘ ’ 1

Fox, J. Weisberg, S. (2011). *An R Companion to Applied Regression*, 2^nd^ Edition. Thousand Oaks CA: Sage
